# HCV serostatus and injection sharing practices among those who obtain syringes from pharmacies and directly and indirectly from syringe services programs in rural New England

**DOI:** 10.1186/s13722-022-00358-7

**Published:** 2023-01-03

**Authors:** Eric Romo, Abby E. Rudolph, Thomas J. Stopka, Bo Wang, Bill M. Jesdale, Peter D. Friedmann

**Affiliations:** 1 Department of Population and Quantitative Health Sciences, University of Massachusetts Chan Medical School, Worcester, MA USA; 2 Department of Epidemiology and Biostatistics, Temple University College of Public Health, Philadelphia, PA USA; 3 Department of Public Health and Community Medicine, Tufts University School of Medicine, Boston, MA USA; 4 Office of Research, University of Massachusetts Chan Medical School-Baystate, Springfield, MA USA

**Keywords:** Rural, Secondary exchange, Syringe services programs, Pharmacy, Hepatitis C virus

## Abstract

**Background:**

Among people who inject drugs (PWID), obtaining syringes via syringe services programs (SSPs) and pharmacies reduces injection sharing practices associated with hepatitis C virus (HCV). Whether indirect use of SSPs via secondary exchange confers a similar benefit remains unknown, particularly in rural settings. We compared HCV serostatus and injection sharing practices by primary syringe source among a sample of rural PWID.

**Methods:**

Data are from a cross-sectional study of adults who use drugs recruited from eleven rural counties in New Hampshire, Vermont, and Massachusetts using respondent-driven sampling (2018–2019). Study staff performed HCV antibody testing. An audio computer-assisted self-interview assessed sociodemographic characteristics, past 30-day injection practices, and past 30-day primary syringe source. Primary syringe source was classified as direct SSP, pharmacy, indirect SSP (secondary exchange), or “other” (friend/acquaintance, street seller, partner/relative, found them). Mixed effects modified Poisson models assessed the association of primary syringe source with HCV seroprevalence and injection sharing practices.

**Results:**

Among 397 PWID, the most common primary syringe source was “other” (33%), then pharmacies (27%), SSPs (22%), and secondary exchange (18%). In multivariable models, compared with those obtaining most syringes from “other” sources, those obtaining most syringes from pharmacies had a lower HCV seroprevalence [adjusted prevalence ratio (APR):0.85, 95% confidence interval (CI) 0.73–0.9985]; however, the upper bound of the 95% CI was close to 1.0. Compared with those obtaining most syringes from other sources, PWID obtaining most syringes directly from SSPs or pharmacies were less likely to report borrowing used syringes [APR(SSP):0.60, 95% CI 0.43–0.85 and APR(Pharmacies):0.70, 95% CI 0.52–0.93], borrowing used injection equipment [APR(SSP):0.59, 95% CI 0.50–0.69 and APR (Pharmacies):0.81, 95% CI 0.68–0.98], and backloading [APR(SSP):0.65, 95% CI 0.48–0.88 and APR(Pharmacies):0.78, 95% CI 0.67–0.91]. Potential inverse associations between obtaining most syringes via secondary exchange and injection sharing practices did not reach the threshold for statistical significance.

**Conclusions:**

PWID in rural New England largely relied on informal syringe sources (i.e., secondary exchange or sources besides SSPs/pharmacies). Those obtaining most syringes from an SSP or pharmacy were less likely to share injection equipment/syringes and had a lower HCV seroprevalence, which suggests using these sources reduces the risk of new HCV infections or serves as proxy for past injection behavior.

**Supplementary Information:**

The online version contains supplementary material available at 10.1186/s13722-022-00358-7.

## Background

The opioid crisis in the United States has fueled a surge in injection drug use and new hepatitis C virus (HCV) infections. Between 2010 and 2019, the number of new HCV infections in the U.S. increased by 387% [1], driven primarily by a rise in injection drug use and related injection practices shown to be associated with acquiring HCV. These include sharing syringes or other injection equipment (e.g., cookers and cottons), and backloading (injecting drugs that someone else prepared, mixed, or divided with a used syringe) [2–4]. In rural communities, new HCV infections have occurred disproportionately among people who inject drugs (PWID) aged ≤ 30 years [5, 6].

In most states, syringe services programs (SSPs) and the nonprescription sale of syringes in pharmacies serve as important interventions to reduce transmission of bloodborne pathogens [7–9]. Studies show reductions in injection sharing practices and HIV transmission among PWID who obtain syringes from SSPs and purchase syringes in pharmacies [10–12], although the evidence for whether using these services reduces HCV transmission remains mixed [10]. Given HCV has greater transmissibility through percutaneous blood exposure compared to HIV, it is unclear whether SSPs and pharmacy syringe sales sufficiently reduce injection sharing practices to a level that lowers the risk of HCV transmission, with some studies suggesting this may depend in part on geographic context [13]. In New England, brick-and-mortar and mobile SSPs are available in some rural areas, but access is often limited by geographic proximity and limited hours of operation, with variation across states.

People who use SSPs commonly extend the indirect reach of these programs through secondary syringe exchange, a practice whereby an individual obtains syringes from an SSP and distributes them to others in their social network [14]. Secondary syringe exchange is common in the urban settings where the phenomenon has been studied [15–17]. However, it remains unclear whether indirect SSP use via secondary exchange offers similar benefits as direct SSP use. Previous studies have not assessed the impact of indirect SSP use on HCV serostatus nor injection sharing practices among rural PWID. Given limited access to formal syringe sources in rural communities [18], it is important to assess whether indirect SSP use among PWID who live in rural parts of the U.S. confers similar reductions in HCV-associated behaviors as it does for those who use SSPs directly.

Using data from the Drug Injection Surveillance and Care Enhancement for Rural Northern New England (DISCERNNE) study, we examined the association of primary syringe source (e.g., SSP, secondary exchange, pharmacy, other sources) with HCV serostatus. A secondary goal was to examine the relationship between primary syringe sources and three injection sharing practices: borrowing used syringes, borrowing used injection equipment, and backloading.

## Methods

### Data source

Data for this investigation were collected as part of the DISCERNNE study, a multi-site, mixed-methods cross-sectional study conducted in 11 rural counties along the Connecticut River Valley in New Hampshire (NH), Vermont (VT), and Massachusetts (MA). The overall goal of DISCERNNE was to characterize the risk environment and epidemiology of overdose and injection-mediated infectious diseases among rural PWID. Further study details and findings from the DISCERNNE study have been published elsewhere [19–21].

### Study participants

Participants were eligible for DISCERNNE if they: (1) were 18 years or older, (2) spent most of the last 30 days living in the study area, (3) used opioids “to get high” (i.e., via any mode of administration) or injected any drug in the last 30 days, and (4) were able to provide written informed consent. We recruited participants at 11 study sites that were chosen after consulting local public health officials, service providers, and harm reduction experts. Study staff recruited participants between May 2018 and October 2019 using respondent-driven sampling (RDS), a chain referral sampling method used to recruit difficult-to-reach populations [22, 23]. Staff recruited RDS seeds (i.e., original participants who serve as initial recruiters) through street outreach and at harm reduction agencies. Once a seed completed the study survey, they were given three unique coupons to refer eligible peers to the study. Recruited peers who participated in the study were given their own set of coupons, and this sampling process continued through multiple waves until the desired sample size was reached. Participants received $10 for each recruited peer who met eligibility criteria and completed the study survey. Participants who successfully recruited 3 eligible peers were offered up to six additional peer-referral coupons. Fifty-one seeds generated a total of 589 survey participants. The present analysis was limited to the 456 participants (77% of total sample) who reported injecting any drug in the past 30 days; individuals were further excluded for having incomplete information on HCV serostatus (n = 31) and primary syringe source (n = 28), resulting in a final analytical sample of 397 PWID. The Baystate Health Institutional Review Board approved the study protocol.

### Data collection

Participants completed a 90-min audio computer-assisted self-interview (ACASI) on touch-screen laptops. The use of ACASI has been shown to enhance perceived confidentiality and increase accurate reporting of sensitive behaviors, including substance use [24, 25]. The ACASI collected self-reported information on sociodemographic characteristics, criminal justice involvement, substance use (lifetime, past 30 days), overdose history, injection and sex behaviors (past 30 days), primary source of syringes (past 30 days), any use of SSPs and pharmacies (past 30 days), substance use treatment (lifetime, past 30 days), healthcare utilization, current mental health, and infectious disease history. Trained staff tested participants for HCV antibodies using the OraQuick HCV rapid antibody test [26]. Participants were compensated $40 for completing the ACASI and infectious disease testing.

## Measures

### Primary syringe source

We assessed participants’ primary syringe source with the question: “During the last 30 days, where have you gotten most of your syringes or needles?” Response options included (1) a “syringe or needle exchange program, in person” (i.e., directly from an SSP), (2) “from someone else who got them from a syringe or needle exchange program” (i.e., secondary exchange), (3) a “pharmacy”, or (4) some other syringe source (a “friend or acquaintance”; “drug dealer or street syringe seller”; “spouse, partner, or relative”; or they “found” their syringes). In our primary analysis, “other” sources served as the reference category. To directly compare those obtaining most syringes from pharmacies with those obtaining most syringes from SSPs on both primary and secondary outcomes, we conducted a supplementary analysis using SSP as the referent category.

### HCV serostatus and injection sharing practices

The primary outcome of interest was HCV serostatus. Participants were considered HCV seropositive if they had a positive rapid HCV antibody test. HCV-RNA testing was not performed. Secondary outcomes included the following past 30-day injection sharing practices: borrowing used syringes (using a syringe known to have been used by someone else), borrowing other used injection equipment (using a cotton, cooker, or water for rinsing/mixing known to have been used by someone else), and backloading (injecting drugs that someone else prepared, mixed, or divided with a used syringe).

### Potential confounding variables

We selected several potentially confounding variables using the disjunctive cause criterion, which proposes that sufficient control for confounding can be achieved by adjusting for variables that cause the exposure, outcome, or both, and excluding variables known to be instrumental variables [27, 28]. Informed by the existing literature [29–35], we controlled for the following variables in our models: age (years); gender (male, female); race (White, non-White); sexual orientation (heterosexual, bisexual/homosexual/other); incarceration within the past 6 months (yes/no); homelessness within the past 6 months (yes/no); years injecting (continuous); injection frequency in the past 30 days (at least once a day, less than daily); injecting multiple times per sitting within the past 30 days (yes/no); injecting heroin (yes/no), cocaine (yes/no), methamphetamine (yes/no), or simultaneous injection of opioid and stimulant (i.e., speedball or screwball) (yes/no) within the past 30 days, and receiving medications for opioid use disorder [MOUD] (ever, never).

### Statistical analysis

We compared participant characteristics, HCV serostatus, and injection sharing practices for those obtaining most syringes directly from SSPs, indirectly through SSPs, from pharmacies, or from other sources using chi-square tests and ANOVA for discrete and continuous variables, respectively. Descriptive statistics were not weighted to account for RDS, and therefore should not be interpreted as population-based estimates. We used multivariable mixed effects modified Poisson regressions to model the relationship between primary syringe source and our primary and secondary outcomes. Mixed effects models accounted for the lack of independence among participants within study sites with a random intercept for study site. Modified Poisson models allow for the direct estimation of prevalence ratios for common binary outcomes [36, 37]. We included all potential confounders described previously in our final multivariable models. We performed a sensitivity analysis using an alternative model building approach; we used bivariate analyses to screen our initial list of potential confounders, including covariates in our final model if they had a bivariate association with the respective outcome at a level of P < 0.10. We analyzed the data using a complete case analysis approach, excluding participants from our regression models if they were missing data on any variables. We assessed multicollinearity using variance inflation factors (VIFs) and did not find it to be a problem as no VIF exceeded 2.0. All analyses were performed using Stata version 14.2 (Stata Corp LP, College Station, TX, USA).

## Results

Participants in the analytic sample were predominantly male (58%), White (93%), injected heroin in the past 30 days (92%), and reported injecting drugs at least daily (59%) in the past 30 days (Table 1). Although not included in Table 1, a majority (63%) reported injecting “street fentanyl or carfentanil powder” in the past 30 days, of whom, 97% also reported injecting heroin. More than half (58%) reported homelessness in the past 6 months. The median age of participants was 33 years (IQR: 28–40) and the median number of years injecting drugs was 9 years (IQR: 5–15). Almost three-quarters of participants (73%) tested positive for HCV antibodies. Nearly one-half (49%) reported borrowing used syringes, while approximately six out of 10 (59%) reported borrowing other used injection equipment, and almost half (48%) reported backloading in the past 30 days.

**Table 1:** Study participant characteristics by primary syringe source: New England (NH, VT, MA), 2018–2019 (n = 397)

Characteristic	Direct SSP (n = 89)	Pharmacy (n = 106)	Indirect SSP (n = 72)	Other Sources (n = 130)
***Sociodemographics-%***				
Female	36	33	44	51
Age (years)-median (IQR)	36 (30–42)	31 (28–39)	34 (28–41)	33 (27–40)
Race: White	92	93	93	92
Sexual orientation: bisexual/homosexual/other^a^	17	16	17	19
High school education or higher	81	69	74	72
Employment: Full/part-time	34	45	29	30
Experienced homelessness (past 6 months)	55	56	57	62
***Criminal justice involvement-%***				
Incarcerated (past 6 months)	23	31	28	39
***Injection drug use-%***				
Years injecting-median (IQR)	9 (4–16.5)	9 (4–14.5)	8 (5–16)	10 (5–14)
Inject at least daily (past 30 days)	75	66	65	46
Inject multiple times per sitting (past 30 days)	81	84	79	73
Inject heroin (past 30 days)	94	94	90	89
Inject cocaine (past 30 days)	52	55	58	47
Inject methamphetamine (past 30 days)	19	25	39	26
Inject speedball or screwball (past 30 days)	37	36	43	29
***Addiction treatment-%***				
Ever received MOUD	76	75	75	73
***Infectious disease-%***				
HCV seropositive	71	69	75	77
***Injection sharing practices-%***				
Borrow used syringes (past 30 days)	34	43	50	65
Borrow other used injection equipment (past 30 days)	39	59	63	69
Backloading (past 30 days)	33	43	50	61

SSP = syringe services program; MOUD = medications for opioid use disorder; HCV = hepatitis C virus Antibody positive

Chi-square tests were used for categorical variables, ANOVA was used for continuous variables

^a^Most PWID in the “bisexual/homosexual/other” category identified as bisexual (88.4%)

With respect to primary syringe source in the past 30 days, 22% reported obtaining most of their syringes directly from an SSP (direct SSP), 27% from a pharmacy, and 18% from someone else who got them from an SSP (indirect SSP). The remaining 33% of participants reported obtaining most of their syringes in the past 30 days from other sources. Among these PWID, primary syringe sources included friends and acquaintances (56%), a drug dealer or street syringe seller (25%), a spouse, partner, or family member (16%), and finding them (3%). A majority of PWID who reported obtaining most syringes directly from SSPs (76%) used no other syringe source; similarly, 86% of those who obtained most syringes from a pharmacy used no other source, and 61% of those who obtained most syringes indirectly from SSPs used no other source. Among those who obtained most syringes from “other” sources, 22% obtained syringes from SSPs (directly or indirectly) or a pharmacy at least once in the past 30 days.

Those obtaining most syringes from “other” sources were more likely to be female than those getting most syringes directly from SSPs, at pharmacies, or indirectly from SSPs (Table 1). Three quarters of those directly using SSPs reported injecting daily, compared with 66% of those who acquired most syringes from pharmacies, 65% of those acquiring syringes indirectly from SSPs, and 46% of those using other sources. Past-month injection of methamphetamine alone was less common than heroin, cocaine, or speedball/screwball injection, but was highest among those obtaining syringes indirectly from SSPs (39%), followed by those getting syringes from other sources (26%), pharmacies (25%), and directly from SSPs (19%). Those obtaining most syringes from other sources were most likely to report borrowing used syringes, borrowing used injection equipment, and backloading, followed by those indirectly acquiring syringes via SSPs, in pharmacies, and directly from SSPs.

In the final mixed effects modified Poisson adjusted model for HCV seroprevalence (Table 2), compared with those getting most syringes from other sources, those who obtained most syringes from pharmacies had a 15% lower HCV seroprevalence (APR: 0.85, 95% CI 0.73–1.00) and those who obtained most syringes directly from SSPs had an 11% lower HCV seroprevalence (APR: 0.89, 95% CI 0.75–1.04) (Table 2). However, the HCV seroprevalence was not statistically significantly different for those who obtained most syringes indirectly via SSPs vs. from other sources.

**Table 2:** Associations between primary syringe source and HCV seroprevalence: New England (NH, VT, MA), 2018–2019

Primary Syringe Source (Past 30 days)	Crude PR (95% CI)	Adjusted PR^a,b^ (95% CI)
Direct SSP	0.92 (0.76–1.12)	0.89 (0.75–1.04)
Pharmacy	0.90 (0.79–1.02)	0.85 (0.73–1.00)
Indirect SSP	0.98 (0.83–1.15)	0.96 (0.82–1.13)
Other Source	reference	reference

*PR* Prevalence ratio; *CI* Confidence interval

^a^Adjusted for age, gender, race, sexual orientation, incarceration, homelessness, years injecting, injection frequency, inject multiple times per sitting, inject heroin, inject cocaine, inject meth, inject speedball/screwball, ever received medication for opioid use disorder

^b^n = 375

In the final multivariable adjusted models for injection sharing practices (Table 3), compared with those who obtained most syringes from other sources, those who obtained most syringes directly from SSPs were significantly less likely to report borrowing used syringes (APR:0.60; 95% CI 0.43–0.85), borrowing used injection equipment (APR:0.59; 95% CI 0.50–0.69), and backloading (APR:0.65; 95% CI 0.48–0.88). Similarly, compared with those who obtained most syringes from “other” sources, those who obtained most syringes from pharmacies were significantly less likely to report borrowing used syringes (APR:0.70; 95% CI 0.52–0.93), borrowing used injection equipment (APR:0.81; 95% CI 0.68–0.98), and backloading (APR:0.78; 95% CI 0.67–0.91). Obtaining most syringes indirectly from SSPs was negatively associated with each injection sharing practice, but these associations were weak and imprecise. When using those who obtained most syringes directly from SSPs as the reference group, those who obtained most syringes from pharmacies were more likely to report borrowing used syringes, borrowing used injection equipment, and backloading. However, only the association with borrowing used injection equipment was statistically significant (APR:1.38; 95% CI 1.17–1.62) (Additional File 1: Table S1).

**Table 3:** Associations between primary syringe source and injection sharing practices: New England (NH, VT, MA), 2018–2019

Primary syringe source (Past 30 days)	Borrowed used syringes (past 30 days)		Borrowed used injection equipment (past 30 days)		Backloading (past 30 days)	
	Crude PR (95% CI)	Adjusted PR^a,b^ (95% CI)	Crude PR (95% CI)	Adjusted PR^a,c^ (95% CI)	Crude PR (95% CI)	Adjusted PR^a,d^ (95% CI)
Direct SSP	0.62 (0.44–0.86)	0.60 (0.43–0.85)	0.63 (0.54–0.73)	0.59 (0.50–0.69)	0.62 (0.48–0.81)	0.65 (0.48–0.88)
Pharmacy	0.70 (0.49–1.01)	0.70 (0.52–0.93)	0.86 (0.69–1.07)	0.81 (0.68–0.98)	0.74 (0.60–0.90)	0.78 (0.67–0.91)
Indirect SSP	0.89 (0.60–1.31)	0.89 (0.62–1.28)	0.96 (0.70–1.32)	0.91 (0.64–1.27)	0.92 (0.69–1.23)	0.92 (0.72–1.17)
Other source	Reference	Reference	Reference	Reference	Reference	Reference

*PR* prevalence ratio; *CI* confidence interval

^a^Adjusted for age, gender, race, sexual orientation, incarceration, homelessness, years injecting, injection frequency, inject multiple times per sitting, inject heroin, inject cocaine, inject meth, inject speedball/screwball, ever received medication for opioid use disorder

^b^n = 372

^c^n = 373

^d^n = 375

We performed a sensitivity analysis using an alternative model building approach (bivariate screening). The results from this approach were not appreciably different from our initial results, with no change in the overall conclusions (Additional File 1: Tables S2 and S3).

## Discussion

In our sample of PWID from rural New England in the United States, we found 22% of participants obtained most of their syringes directly from an SSP, 27% from a pharmacy, 18% indirectly from an SSP via secondary syringe exchange, and the remaining 33% from other sources. Compared with the use of other sources, indirect SSP use was not meaningfully associated with HCV seroprevalence and was only weakly and imprecisely associated with a lower prevalence of injection sharing practices. In contrast, obtaining most syringes from pharmacies or directly from SSPs were each modestly associated with lower HCV seroprevalence and strongly associated with a lower risk of borrowing used syringes, borrowing used injection equipment, and backloading.

Secondary syringe exchange was relatively common in our sample of rural PWID. It is difficult to compare the prevalence of secondary exchange in our study with that of previous studies since those engaging in secondary exchange were defined differently across studies. However, the proportion of PWID in our study who *exclusively* obtained syringes via secondary exchange (11%) was much higher than that reported in previous studies among urban PWID (1.4–4.3%) [15, 38]. Urban PWIDs’ greater access to SSPs could explain this difference [18], as they may not need to rely as frequently on secondary exchange to acquire clean syringes. Research shows that urban participants who acquire syringes via secondary exchange cite inconvenient locations and hours as major barriers to direct SSP use [39]. Scarcity in terms of limited local access and limited hours of operation at formal SSPs may be more salient to the use of secondary syringe exchange in rural areas. At the time of this study, only five brick-and-mortar SSPs were operating in the 11-county study region: four across six VT counties, one in the sole MA county, and zero across four NH counties. Although the SSP in the rural MA county had operating hours comparable to SSPs in larger MA cities, the four SSPs in VT were only open once or twice per week for a total of three to eight hours. (In contrast, the SSP in VT’s largest city is open five days a week for a total of 35 h.) In terms of pharmacy syringe access, MA, NH, and VT all permit the nonprescription sale of syringes in pharmacies, but participation is optional. Although nonprescription syringe sales are available in nearly all MA pharmacies [40], recent qualitative research suggests PWID in rural NH experience very limited in-state pharmacy syringe access [41]. Given the majority (59%) of PWID in our study reported injecting drugs at least daily, having a clean syringe for each injection would require regular access to a large number of syringes. Our findings suggest that secondary syringe exchange is an important source of clean syringes among rural PWID in northern New England.

Although indirect use of SSPs was not associated with HCV seroprevalence, pharmacy use and direct SSP use were modestly correlated with lower HCV seroprevalence compared with using “other” syringe sources. Rural PWID who obtained most of their syringes from a pharmacy had a 15% lower prevalence of HCV antibodies compared with those who obtained most syringes from other sources; however, the upper bound of the 95% confidence interval was very close to 1.0, which suggests our findings may also be compatible with no significant difference. Previous reviews and meta-analyses have found inconsistent evidence that pharmacy syringe sales or SSPs reduce HCV incidence or prevalence [10, 42–44]. The literature is especially sparse for pharmacy syringe sales, with very few studies including HCV incidence or prevalence as an outcome [44]. Our study adds to the existing literature by examining the relationship between primary syringe source and HCV seroprevalence in a rural setting. While not definitive, our results suggest that obtaining syringes from a pharmacy or SSP may reduce the risk of HCV infection among rural PWIDs. However, since HCV seroprevalence alone (measured via antibodies) cannot differentiate between incident, prevalent, or resolved infection, we cannot ascertain the temporal relationship between exposure and disease. It is possible that participants were infected with HCV before they began obtaining syringes from their reported primary source.

Consistent with a large body of research in urban settings [10, 42, 43], we observed that acquiring most syringes from pharmacies or directly through SSPs was associated with a lower risk of borrowing used syringes and other injection equipment, and backloading among rural PWID. Obtaining most syringes directly from an SSP appears to have a greater impact on injection sharing practices than obtaining most syringes from a pharmacy. Compared to obtaining most syringes directly from an SSP, obtaining most syringes from a pharmacy was associated with a 16% higher risk of borrowing used syringes and a 38% higher risk of borrowing used injection equipment, although only the latter was statistically significant. This makes sense, given that SSPs and pharmacies both provide syringes, but only SSPs provide other injection equipment (e.g., cotton filters, cookers, rinse water) as well as harm reduction education, testing, and referrals. Additionally, although direct SSP use and pharmacy use were similarly associated with borrowing used syringes, syringes are typically available at no cost from SSPs, whereas they must be purchased from pharmacies. This may result in greater access to sterile syringes for those who obtained most syringes from SSPs. In our rural sample, the sociodemographic characteristics of those who obtained most of their syringes from pharmacies were not significantly different from those who obtained most of their syringes from SSPs. Of note, other studies in urban settings have reported differences in sociodemographic characteristics by primary syringe source [45, 46]. It is possible that in urban settings, the demographics are more heterogeneous, so differences are more likely to be observed. For example, the sample in our study is fairly homogeneous in terms of race and ethnicity.

Although the associations observed between acquiring most syringes through secondary exchange (vs. other sources) and each of the injection sharing practices were in the anticipated direction, these associations were weak and imprecise. These results differ from two previous studies conducted in three urban U.S. cities, which observed stronger associations between indirect SSP use and injection sharing practices [15, 38]. Several factors may explain the differences between our findings and those of previous studies. The first study, conducted in Chicago, used a more inclusive definition for indirect SSP use. Our study defined indirect SSP use as obtaining *most* syringes in the past 30 days from someone else who got them from an SSP. In contrast, the Chicago study defined their group of “mixed/secondary exchangers” as PWID who, in the past 6 months, obtained at least *some* needles indirectly from an SSP through other persons [15]. Only 5% of these mixed/secondary exchangers exclusively obtained syringes via secondary exchange (compared with 61% of those in our analysis), with the remaining 95% reporting getting syringes directly from an SSP at least once in the past 6 months. This level of mixed indirect and direct SSP use may have accounted for the strong negative association observed between mixed/secondary exchange and borrowing used syringes. In the second study, the authors explain their results may not be generalizable to other PWID populations given their study was conducted in an area with an unconventional SSP that delivered syringes directly to clients’ homes with no limit on the number of syringes exchanged [38]. Given the increased opportunity for direct SSP use, PWID relying on secondary exchange may have had better access to SSP clients, thus increasing their likelihood of fully meeting their syringe needs via secondary exchange.

Nevertheless, our findings should not be interpreted to mean that secondary syringe exchange should be discouraged in rural settings. First, strong evidence supports that secondary exchange increases the number of people served by SSPs by reaching PWID who are unable or unwilling to attend an SSP in person. In previous U.S. studies in urban settings, PWID who participated in secondary exchange reported fear of exposure, fear of police harassment, and inconvenient SSP locations and hours as major barriers to attending SSPs [14, 39]. Secondary exchange through one’s peer network effectively overcomes these barriers. Indeed, in our analysis, compared with those getting syringes directly from SSPs, those using SSPs indirectly were more likely to be female or to have been recently incarcerated, two populations shown to have fears of negative consequences from SSP participation [47–50]. Second, existing secondary exchange networks could serve as the basis for peer-based interventions among rural PWID. PWID who obtain syringes via secondary exchange do not receive the many ancillary services available at SSPs, which may explain why direct SSP use was strongly associated with reduced injection sharing practices while indirect SSP use was not. Therefore, training peer exchangers to deliver harm reduction messages and some of the ancillary services (e.g., facilitate referrals) might enhance the effectiveness of secondary exchange to reduce injection sharing practices and HCV prevalence. Peer-based interventions leveraging existing secondary exchange networks have been shown to be effective in Canada, the Russian Federation, and China [51–53].

This study has several limitations. First, the data are cross-sectional, which precludes statements about causality. Second, as previously mentioned, the study did not include HCV-RNA testing, so our seroprevalence data cannot differentiate between incident, prevalent, or resolved infection. The relatively high HCV seroprevalence (73%) in our sample suggests many of these cases may have developed long before we measured participants’ primary syringe source, and perhaps even before SSPs or nonprescription pharmacy syringe sales were available in the study area. Therefore, the modest and non-significant associations across primary syringe sources do not necessarily mean that those currently obtaining syringes from SSPs and pharmacies will not be less likely to develop a new HCV infection in the future. Given SSP and pharmacy use were negatively associated with injection sharing practices, we may have observed stronger protective associations between these syringe sources and HCV had we been able to focus on incident infections. Future research among rural PWID should include HCV-RNA testing to better assess the relationship between syringe source and HCV infection. Third, given the sample size, the analysis had limited power to detect differences in associations across the primary syringe source groups. Fourth, the self-reported data for primary syringe source creates the potential for social desirability bias. Previous research suggests, however, the use of ACASI may diminish socially desirable responses to sensitive questions [54].

Our measure of participants’ primary syringe source over the past 30 days is also subject to misclassification. For instance, although we classified friends, spouses, relatives and street sellers as “other” syringe sources, it is possible the PWID in our sample did not know the original source of the syringes obtained from others. If many of these syringes originally came from SSPs, then we have underestimated the number of individuals acquiring syringes indirectly via SSPs in our sample. However, from the perspective of risk perception, a participant truly not knowing the original source of their syringe remains distinct from a participant knowing a peer is giving them a sterile syringe that was obtained from an SSP. In the former scenario, the participant is assuming greater risk of infection than the participant who knowingly obtains most syringes via secondary exchange.

Finally, although we used RDS for recruitment, because several key assumptions were not met, we did not use RDS weights [22, 55]. Therefore, as explained in the methods, our sample should be treated as a convenience rather than a representative sample. Despite these limitations, this analysis adds to the relatively small body of research on secondary syringe exchange and is among the first conducted among rural PWID in the U.S., a high-risk and understudied population.

## Conclusion

Secondary syringe exchange is a common practice among PWID in the rural northeastern U.S. However, indirect SSP use was not associated with HCV seroprevalence and only weakly associated with injection sharing practices. Further research is needed to determine whether existing syringe exchange networks can be leveraged to deliver peer-based harm reduction interventions to rural PWID. Finally, these findings reaffirm the important role SSPs and pharmacies play in reducing injection sharing practices, and suggest their benefits might extend to reducing the risk of future HCV infections.

## Supplementary Information

**Additional file 1: Table S1.** Associations between primary syringe source and injection sharing practices, with direct SSP as the reference group. **Table S2**. Associations between primary syringe source and HCV seroprevalence using an alternative model building approach (bivariate screening). **Table S3**. Associations between primary syringe source and injection sharing practices using an alternative model building approach (bivariate screening). **Table S4**. Syringe sources used at least once in the past 30 days by primary syringe source (past 30 days). **Table S5**. Frequency of participants whose primary syringe source was their only source in the past 30 days. **Table S6**. Sensitivity analysis - Associations of syringe source with HCV serostatus and injection sharing practices using alternative categories for syringe source.

## Data Availability

All data generated and analyzed during the current study are available from the corresponding author on reasonable request.

## Abbreviations

HCV: Hepatitis C virus
PWID: People who inject drugs
SSP: Syringe services program
DISCERNNE: Drug Injection Surveillance and Care Enhancement for Rural Northern New England
NH: New Hampshire
VT: Vermont
MA: Massachusetts
RDS: Respondent-driven sampling
ACASI: Audio computer-assisted self-interview
MOUD: Medications for opioid use disorder
