# Supplementary material for: HCV serostatus and injection sharing practices among those who obtain syringes from pharmacies and directly and indirectly from syringe services programs in rural New England
Source: Addict Sci Clin Pract. 2023 Jan 3;18:2. doi: 10.1186/s13722-022-00358-7 (PMC9809047; doi:10.1186/s13722-022-00358-7)
Supplement: Supplementary file 1 — Additional file 1: Table S1. Associations between primary syringe source and injection sharing practices, with direct SSP as the reference group. Table S2. Associations between primary syringe source and HCV seroprevalence using an alternative model building approach (bivariate screening). Table S3. Associations between primary syringe source and injection sharing practices using an alternative model building approach (bivariate screening). Table S4. Syringe sources used at least once in the past 30 days by primary syringe source (past 30 days). Table S5. Frequency of participants whose primary syringe source was their only source in the past 30 days. Table S6. Sensitivity analysis - Associations of syringe source with HCV serostatus and injection sharing practices using alternative categories for syringe source. [file 13722_2022_358_MOESM1_ESM.docx]

**Table S1.** Supplementary Analysis – Associations between primary syringe source and injection sharing practices, with direct SSP as the reference group: New England (NH, VT, and MA), 2018-2019

|  | Borrowed used syringes  (past 30 days) | | Borrowed used injection equipment  (past 30 days) | | Backloading  (past 30 days) | |
| --- | --- | --- | --- | --- | --- | --- |
| Primary Syringe Source  (Past 30 days) | Crude PR  (95% CI) | Adjusted PR^a^  (95% CI) | Crude PR  (95% CI) | Adjusted PR^b^  (95% CI) | Crude PR  (95% CI) | Adjusted PR^c^  (95% CI) |
| Pharmacy | 1.13  (0.69-1.85) | 1.16  (0.73-1.82) | 1.36  (1.12-1.66) | 1.38  (1.17-1.62) | 1.18  (0.93-1.50) | 1.21  (0.95-1.54) |
| Indirect SSP | 1.44  (1.00-2.06) | 1.47  (0.96-2.25) | 1.52  (1.10-2.11) | 1.53  (1.10-2.13) | 1.48  (1.05-2.09) | 1.42  (0.98-2.05) |
| Other Source | 1.62  (1.16-2.26) | 1.66  (1.17-2.34) | 1.59  (1.37-1.84) | 1.69  (1.44-1.98) | 1.60  (1.24-2.07) | 1.55  (1.14-2.10) |
| Direct SSP | reference | reference | reference | reference | reference | reference |

Note: PR=prevalence ratio; CI=confidence interval  ^a^Adjusted for age, gender, race, sexual orientation, incarceration, homelessness, years injecting, injection frequency, inject multiple times per sitting, inject heroin, inject cocaine, inject meth, inject speedball/screwball, ever received medication for opioid use disorder ^b^n = 372; ^c^n = 373; ^d^n = 375

**Table S2.** Sensitivity Analysis – Associations between primary syringe source and HCV seroprevalence using an alternative model building approach^a^: New England (NH, VT, and MA), 2018-2019 (n=381)

| Primary Syringe Source  (Past 30 days) | Crude PR  (95% CI) | Adjusted PR^b^  (95% CI) |
| --- | --- | --- |
| Direct SSP | 0.92  (0.76-1.12) | 0.88  (0.75-1.02) |
| Pharmacy | 0.90  (0.79-1.02) | 0.87  (0.76-0.99) |
| Indirect SSP | 0.98  (0.83-1.15) | 0.97  (0.83-1.14) |
| Other Source | reference | reference |

Note: PR=prevalence ratio; CI=confidence interval

^a^Covariates were included in the final model if they had a bivariate association with the respective outcome at a level of P < 0.10

^b^Adjusted for age, race, sexual orientation, homeless, incarceration, years injecting, injection frequency, inject multiple times per sitting, ever received MOUD

**Table S3.** Sensitivity Analysis – Associations between primary syringe source and injection sharing practices using an alternative model building approach^a^: New England (NH, VT, and MA), 2018-2019

|  | Borrowed used syringes  (past 30 days) | | Borrowed used injection equipment  (past 30 days) | | Backloading  (past 30 days) | |
| --- | --- | --- | --- | --- | --- | --- |
| Primary Syringe Source  (Past 30 days) | Crude PR  (95% CI) | Adjusted PR^a^  (95% CI) | Crude PR  (95% CI) | Adjusted PR^b^  (95% CI) | Crude PR  (95% CI) | Adjusted PR^c^  (95% CI) |
| Direct SSP | 0.62  (0.44-0.86) | 0.61  (0.44-0.85) | 0.63  (0.54-0.73) | 0.62  (0.55-0.70) | 0.62  (0.48-0.81) | 0.61  (0.54-0.70) |
| Pharmacy | 0.70  (0.49-1.01) | 0.70  (0.51-0.97) | 0.86  (0.69-1.07) | 0.84  (0.69-1.02) | 0.74  (0.60-0.90) | 0.83  (0.71-0.98) |
| Indirect SSP | 0.89  (0.60-1.31) | 0.86  (0.56-1.31) | 0.96  (0.70-1.32) | 0.91  (0.66-1.27) | 0.92  (0.69-1.23) | 0.91  (0.64-1.31) |
| Other Source | reference | reference | reference | reference | reference | reference |

Note: PR=prevalence ratio; CI=confidence interval

^a^Covariates were included in the final model if they had a bivariate association with the respective outcome at a level of P < 0.10

^b^Adjusted for sexual orientation, homelessness, inject multiple times per sitting, inject cocaine, inject methamphetamine, inject speedball/screwball (n=385)
^c^Adjusted for sexual orientation, homelessness, incarceration, inject multiple times per sitting, inject methamphetamine, inject speedball/screwball (n=386)
^d^Adjusted for gender, sexual orientation, homelessness, incarceration, inject multiple times per sitting, inject cocaine, inject methamphetamine, inject speedball/screwball, ever received MOUD (n=384)

**Table S4.** Syringe sources used at least once in the past 30 days by primary syringe source (past 30 days). New England (NH, VT, MA), 2018-2019

|  | Primary Syringe Source (Past 30 days) | | | | |
| --- | --- | --- | --- | --- | --- |
| Syringe Sources Used At Least Once in Past 30 days | | Direct SSP (n=89) | Pharmacy (n=106) | Indirect SSP (n=72) | Other Sources (n=130) |
| SSP, in person (n=118) | | 89 (100%) | 7 (7%) | 12 (17%) | 10 (8%) |
| Pharmacy (n=147) | | 13 (15%) | 106 (100%) | 10 (14%) | 18 (14%) |
| From someone else who got them from an SSP (n=104) | | 12 (13%) | 4 (4%) | 72 (100%) | 16 (12%) |
| Other sources: (n=178) | | 15 (17%) | 10 (9%) | 23 (32%) | 130 (100%) |

Note: Percentages are column percentages

**Table S5.** Frequency of participants whose primary syringe source was their only source in the past 30 days. New England (NH, VT, MA), 2018-2019

| Primary Syringe Source  (Past 30 days) | Primary syringe source was the only syringe source in the past 30 days – no. (%) |
| --- | --- |
| Direct SSP (n=89) | 68 (76%) |
| Pharmacy (n=106) | 91 (86%) |
| Indirect SSP (n=72) | 44 (61%) |
| Other Sources (n=130) | 102 (78%) |

**Table S6.** Sensitivity analysis - Associations of syringe source with HCV serostatus and injection sharing practices using alternative categories for syringe source. New England (NH, VT, and MA), 2018-2019

| Outcome | Syringe Source (Past 30 days) | Crude PR  (95% CI) | Adjusted PR^a^  (95% CI) |
| --- | --- | --- | --- |
| HCV seropositive | Any direct SSP use ^b^ | 0.95 (0.78-1.16) | 0.92 (0.79-1.07) |
|  | Most syringes from Pharmacy | 0.93 (0.81-1.08) | 0.91 (0.78-1.05) |
|  | Most syringes via indirect SSP use | 0.97 (0.84-1.12) | 0.96 (0.83-1.11) |
|  | Most syringes Other Source | reference | reference |
| Borrow used syringes | Any direct SSP use ^b^ | 0.73 (0.53-1.01) | 0.71 (0.53-0.96) |
|  | Most syringes from Pharmacy | 0.72 (0.52-1.02) | 0.73 (0.58-0.91) |
|  | Most syringes via indirect SSP use | 0.83 (0.49-1.39) | 0.85 (0.52-1.37) |
|  | Most syringes Other Source | reference | reference |
| Borrowed other used | Any direct SSP use ^b^ | 0.70 (0.57-0.86) | 0.64 (0.53-0.77) |
| injection equipment | Most syringes from Pharmacy | 0.85 (0.69-1.06) | 0.83 (0.69-0.99) |
|  | Most syringes via indirect SSP use | 0.96 (0.70-1.32) | 0.92 (0.67-1.26) |
|  | Most syringes Other Source | reference | reference |
| Backloading | Any direct SSP use ^b^ | 0.71 (0.50-1.01) | 0.72 (0.52-1.00) |
|  | Most syringes from Pharmacy | 0.76 (0.59-0.99) | 0.82 (0.68-0.99) |
|  | Most syringes via indirect SSP use | 0.93 (0.62-1.39) | 0.94 (0.65-1.34) |
|  | Most syringes Other Source | reference | reference |

Note: PR=prevalence ratio; CI=confidence interval

^a^Adjusted for age, gender, race, sexual orientation, incarceration, homelessness, years injecting, injection frequency, inject multiple times per sitting, inject heroin, inject cocaine, inject meth, inject speedball/screwball, ever received medication for opioid use disorder

^a^This group included participants who obtained most of their syringes from a source other than an SSP (i.e., pharmacy, indirect SSP use, other source)
